# Analogue cosmological particle creation in an ultracold quantum fluid of light

**DOI:** 10.1038/s41467-022-30603-1

**Published:** 2022-05-25

**Authors:** Jeff Steinhauer, Murad Abuzarli, Tangui Aladjidi, Tom Bienaimé, Clara Piekarski, Wei Liu, Elisabeth Giacobino, Alberto Bramati, Quentin Glorieux

**Affiliations:** 1 Laboratoire Kastler Brossel, Sorbonne Université, CNRS, ENS-PSL Research University, Collège de France, Paris, 75005 France; 2 Department of Physics, Technion—Israel Institute of Technology, Technion City, Haifa, 32000 Israel

**Keywords:** Single photons and quantum effects, Cosmology

## Abstract

The rapid expansion of the early universe resulted in the spontaneous production of cosmological particles from vacuum fluctuations, some of which are observable today in the cosmic microwave background anisotropy. The analogue of cosmological particle creation in a quantum fluid was proposed, but the quantum, spontaneous effect due to vacuum fluctuations has not yet been observed. Here we report the spontaneous creation of analogue cosmological particles in the laboratory, using a quenched 3-dimensional quantum fluid of light. We observe acoustic peaks in the density power spectrum, in close quantitative agreement with the quantum-field theoretical prediction. We find that the long-wavelength particles provide a window to early times. This work introduces the quantum fluid of light, as cold as an atomic Bose-Einstein condensate.

## Abstract (web summary)

Under certain conditions light can act as a fluid like a Bose-Einstein condensate. Here the authors discuss an analogy of cosmological particle creation using such a quantum fluid of light.

## Introduction

The expansion of a universe stretches all length scales, including the wavelengths of the particle modes. Thus, the frequencies of the modes evolve with time^1^, which implies that the modes at early and late times are related by a Bogoliubov transformation^2–6^. This field theory approach avoids the microscopic details, and predicts the spontaneous production of cosmological particles, including the primordial density fluctuations which led to the acoustic peaks in the cosmic microwave background (CMB) spectrum^4,6,7^. It is particularly relevant since the acoustic peaks can be described by linear perturbation theory^8^.

The field theory approach inspired the subject of analogue cosmological particle creation, in which laboratory experiments mimic the dynamics of scalar fields in curved space times^9–15^. The experiments even allow for measurement over time, which is impossible in the real universe, for which there is only one time of observation. Since the model is independent of the microscopic description of the medium, various quantum fluids were proposed for the study of cosmological particle creation in analogue universes^9–15^. In a two-dimensional atomic Bose-Einstein condensate, a qualitative comparison with cosmological particle creation was reported^16^. In a 1-dimensional experiment not related to quantum fluids, a rapid switch in the trapping field of two ions led to phonon pair creation and formation of spatial entanglement^17^.

Analogue cosmological particle creation is a type of dynamical Casimir effect^18–20^, which was observed in a superconducting circuit^21^ and an optical fiber^22^. The classical, stimulated version of the effect was reported in a Bose-Einstein condensate^23^, but the observation of the quantum effect in a quantum fluid has not been reported. Pairs can also be produced by a modulational instability^24^.

**Fig. 1:**
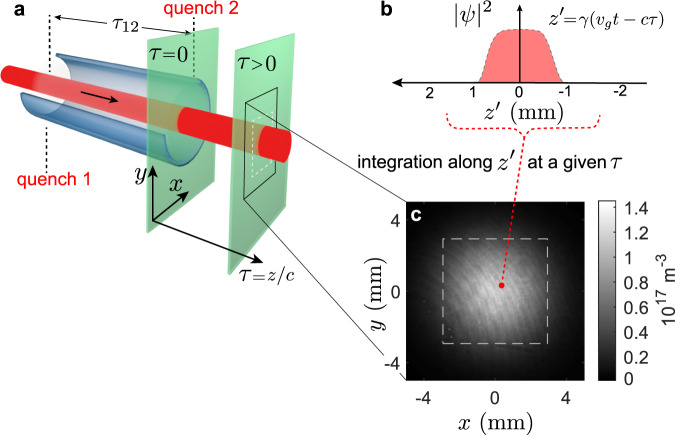
The analogue universe. **a** The fluid of light (red) is a laser pulse traversing a heated ^85^Rb vapor cell. The axial position gives the effective time *τ*. The quenches occur at the entrance and exit of the vapor cell. *τ* = 0 corresponds to quench 2. The time between the two quenches is *τ*_12_. **b** The true time gives an effective third spatial dimension *z*′. **c** Typical image of the fluid of light integrated along *z*′, given in units of photon density. An effective time *τ* = 103 ps after quench 2 is shown.

We simulate expanding and contracting universes in a 3-dimensional quantum fluid of light, as coherent as an atomic Bose-Einstein condensate, and we observe time-resolved analogue cosmological particle creation out of vacuum fluctuations. Our quantum fluid is a near-resonant laser pulse traversing a warm atomic vapor cell, as illustrated in Fig. 1a. Within the vapor cell, the repulsive interactions between photons are mediated by the atoms, due to Kerr nonlinearity induced by the atomic resonance^25^. The interactions are suddenly quenched to zero when the laser beam exits the vapor cell^26^. This configuration mimics an expanding universe, since a rapid reduction of the interactions causes a sudden red shift of the energy spectrum^9–13^. We also observe the reverse process at the cell entrance, in which the interaction suddenly appears, mimicking a contracting universe. We demonstrate that both processes produce pairs of analogue cosmological particles, which confirms the predictions of Ref. ^26^.

## Results

### Theoretical techniques

Our approach relies on the analogy between light propagation in a Kerr nonlinear medium, and the temporal dynamic of an atomic Bose-Einstein condensate. The effective time is $\tau =z/c$, where $z$ is the position in the direction of propagation, and $c$ is the speed of light. This effective time is equivalent to true time for the sake of quantum mechanical quasiparticle creation^26,27^. With no approximation other than the usual paraxial and slowly-varying envelope approximations^28^, we extend the standard monochromatic limit^28^ and find that our fluid is described by the 3-dimensional Gross-Pitaevskii equation

1 $$i{{\hslash }}\frac{\partial \psi }{\partial \tau }=-\frac{{{{\hslash }}}^{2}}{2m}{\nabla }^{2}\psi +U({{{{{\bf{r}}}}}},\tau)\psi +g({{{{{\bf{r}}}}}},\tau){|\psi|}^{2}\psi$$

where $\psi$ is the slowly-varying envelope of the electric field, ${\left|\psi \right|}^{2}$ is the volume density of the photons, $m$ is their effective mass, $U$ is an external potential, and $g{\left|\psi \right|}^{2}$ is the mean-field interaction energy. The three spatial dimensions of $\nabla$ correspond to the transverse coordinates $(x,y)$ and to ${z}^{{\prime} }=\gamma ({v}_{{{{{{\rm{g}}}}}}}t-c\tau )$, which is a coordinate comoving with the laser pulse at the group velocity ${v}_{{{{{{\rm{g}}}}}}}$, and compressed by the factor $\gamma ={(-{v}_{{{{{{\rm{g}}}}}}}^{2}{k}_{0}{D}_{0})}^{-1/2}$, where ${k}_{0}$ is the wavenumber of the laser, and ${D}_{0}$ is the group velocity dispersion (see 3-dimensional Gross-Pitaevskii equation in Supplementary Methods). In other words, a laser pulse viewed in the ${z}^{{\prime} }$ coordinate would appear stationary and compressed relative to its length in the $z$ coordinate.

We study the analogue cosmological particles using the static structure factor, in analogy with the CMB power spectrum. The static structure factor has been used to study density fluctuations in Bose-Einstein condensates^16,29^, and we apply this technique to a fluid of light. It is given by $S({{k}_{x}},{{k}_{y}},{{k}_{z^{\prime}}})=\langle {|\delta \rho ({{k}_{x}},{{k}_{y}},{{k}_{z^{\prime}}})|}^{2}\rangle/M$, where $\delta \rho ({{k}_{x}},{{k}_{y}},{{k}_{z^{\prime}}})$ is the spatial Fourier transform of the density fluctuation at time $\tau$, and $M$ is the total number of particles in the fluid. With this definition, a zero-temperature, non-interacting gas has $S(k)$ = 1, reflecting the presence of spatial shot noise. The operator ${\hat{b}}_{{{{{{\bf{k}}}}}}}^{{{\dagger}} }$ corresponds to the creation of a quasiparticle after the quench, in mode ${{{{{\bf{k}}}}}}=({{k}_{x}},{{k}_{y}},{{k}_{z^{\prime}}})$ oscillating at frequency ${\omega }_{k}$. In the presence of quasiparticle populations $N\equiv \langle {\hat{b}}_{{{{{{\bf{k}}}}}}}^{{{\dagger}} }{\hat{b}}_{{{{{{\bf{k}}}}}}}\rangle$ and correlations $C\equiv \langle {\hat{b}}_{{{{{{\bf{k}}}}}}}{\hat{b}}_{-{{{{{\bf{k}}}}}}}\rangle$, the static structure factor within the Bogoliubov approximation is given by (see $S(k)$ including absorption in Supplementary Methods)

2 $$S\left(k\right)=1+2N+2{{{{{\rm{Re}}}}}}\left(C{e}^{-i2{\omega }_{k}\tau }\right).$$

The populations and correlations are given by

3 $$N={\beta }^{2}+{N}_{0}\left({\alpha }^{2}+{\beta }^{2}\right)+2\alpha \beta {{{{{\rm{Re}}}}}}\left({C}_{0}\right)$$

4 $$C=\alpha \beta +{C}_{0}{\alpha }^{2}+{C}_{0}^{* }{\beta }^{2}+2\alpha \beta {N}_{0}$$

where ${N}_{0}\equiv \langle {\hat{a}}_{{{{{{\bf{k}}}}}}}^{{{\dagger}} }{\hat{a}}_{{{{{{\bf{k}}}}}}}\rangle$ and ${C}_{0}\equiv \left\langle {\hat{a}}_{{{{{{\bf{k}}}}}}}{\hat{a}}_{-{{{{{\bf{k}}}}}}}\right\rangle$ are the populations and correlations before the quench, respectively,$\,{\hat{a}}_{{{{{{\bf{k}}}}}}}^{{{\dagger}} }$ corresponds to the creation of a quasiparticle before the quench, and the operators are related by the Bogoliubov transformation ${\hat{b}}_{{{{{{\bf{k}}}}}}}=\alpha {\hat{a}}_{{{{{{\bf{k}}}}}}}+\beta {\hat{a}}_{{{{{{\boldsymbol{-}}}}}}{{{{{\bf{k}}}}}}}^{{{\dagger}} }$. For our series of two quenches, Eqs. (3) and (4) are applied twice. Since each quench either starts or ends with no interactions, α and β are the same Bogoliubov coefficients which diagonalize the Hamiltonian of a weakly-interacting quantum fluid (see $S(k)$ including absorption in Supplementary Methods). In the absence of quasiparticles before a given quench, the pair production is spontaneous, and Eqs. (3) and (4) become $N={\beta }^{2}$ and $C=\alpha \beta$. On the other hand, a distribution of quasiparticles before the quench, thermal or otherwise, will stimulate additional pairs.

### Experimental design

To create the fluid of light, we use a laser pulse with a 4 mm Gaussian waist and a power of 100 mW, propagating in an ^85^Rb vapor cell heated to 150 °C. A pulse length of 100 ns is employed to avoid saturating the camera used for observation. The laser is detuned −1.5 GHz (90 natural linewidths, 6 times the Doppler broadening) from the D2 $5{S}_{1/2},{F}=3\to 5{P}_{3/2}$ transition, giving ${v}_{{{{{{\rm{g}}}}}}}$ = 0.007$c$. The interaction energy and healing length $\xi$ = 60 µm are determined by the nonlinear change in the refractive index $\triangle n$, which is computed from the experimental parameters (see Determination of $\triangle n$ in Methods). By taking into account the compression factor $\gamma$, this configuration leads to a weakly interacting photon gas with a thickness of 2 mm in the $z^{\prime}$ coordinate, and a dimensionless interaction coefficient $\rho {a}_{{{{{{\rm{s}}}}}}}^{3}$ = 7 × 10^−14^, where $\rho$ is the average photon density, and ${a}_{{{{{{\rm{s}}}}}}}$ is the effective scattering length.

The fluid of light is imaged on a sCMOS camera, as shown in Fig. 1c. We tune the imaging system to pick out a certain $z$ after the cell (fixing the effective time $\tau$ after the second quench), and the camera integrates over true time (thus integrating over $z$’), as illustrated in Fig. 1b. According to the Fourier slice theorem, this integration in position space gives a slice in $k$-space^30^. Thus, an ensemble of 200 images is obtained for each $\tau$, and the power spectrum $S({k}_{x},{k}_{y},{k}_{z{\prime}}=0)$ is computed by 2-dimensional Fourier transforms within the dashed square shown in Fig. 1c. The computation partially removes the effects of any drifts such as thermal convection, and accounts for the measured quantum efficiency of the camera (see Computation of $S({k}_{x},{k}_{y},{k}_{z{\prime}}=0)$ in Methods).

### Observation of analogue cosmological particle creation

**Fig. 2:**
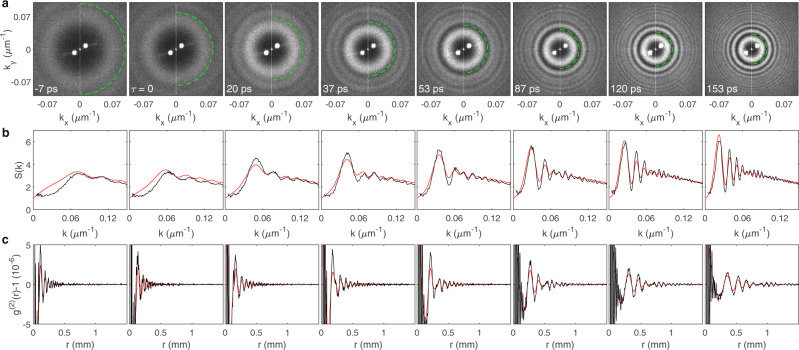
Analogue cosmological particle creation in a quantum fluid of light. **a** The static structure factor *S*(*k*_*x*_, *k*_*y*_, *k*_*z*′_ = 0) at various times after the second quench. The dashed green curves indicate the first minimum of the red curves in (**b**). The symmetric white points near the center of all panels are due to spurious fringes in the imaging system. **b** Radial profiles of (**a**). The black curves are the experimental data. The red curves are the prediction for analogue cosmological particle creation, from Eq. (2). **c** Density-density correlations. The experimental (black) and theoretical (red) curves are obtained from **b** by the spherical Fourier transform of Eq. (5).

In Fig. 2a we observe ring patterns in $S({k}_{x},{k}_{y},{k}_{z{\prime} }=0)$, oscillating as a function of $k$. These oscillations are the experimental signature of analogue cosmological particle creation, in close analogy with the acoustic peaks in the angular spectrum of the CMB. Pairs of quasiparticles with momenta $\pm {{{{{\bf{k}}}}}}$ are generated at the moment of the quench with a random overall phase, but a definite phase relationship between $+{{{{{\bf{k}}}}}}$ and $-{{{{{\bf{k}}}}}}$, and oscillate with various frequencies ${\omega }_{k}$. Only certain $k$-values interfere constructively at the observation time $\tau$, resulting in a ring pattern. The rings shrink with $\tau$ since lower frequencies take longer to develop oscillations. The shrinking pattern of rings is described quantitatively by Eq. (2). The radius of the first minimum in Fig. 2a is seen to be in good agreement with the theoretical prediction of Eq. (2), indicated by the dashed green curve. The azimuthal averages $S(k)$ of $S({k}_{x},{k}_{y},{k}_{z{\prime} }=0)$ are indicated in black in Fig. 2b. The red curves are calculated from Eq. (2), taking into account the two quenches, and the variations in $\alpha$, $\beta$, and ${\omega }_{k}$ which result from the measured absorption (see $S(k)$ including absorption in Supplementary Methods). Very good agreement between the experimental black and theoretical red curves is seen.

We also determine the spatial density correlations produced by the analogue cosmological particle creation. We derive the density-density correlation function ${g}^{(2)}(r)$ from $S\left(k\right)$ by the 3-dimensional spherically-symmetric Fourier transform

5 $${g}^{(2)}(r)-1=\frac{1}{2{\pi }^{2}\rho }\int {dk}\,{k}^{2}\frac{{{\sin }}\left({kr}\right)}{{kr}}\left[S\left(k\right)-1\right].$$

Figure 2c shows ${g}^{(2)}(r)-1$, found by applying Eq. (5) to Fig. 2b. The oscillations are spherical shells propagating outward. The correlations are seen to reach increasing distances as time increases. They are on the order of 10^−6^, which implies that the relative density fluctuations are on the order of 10^−3^. The oscillations are clear despite the small signal, due to the high sensitivity of the optical detection. The theoretical red curves are obtained by applying Eq. (5) to Eq. (2), and quantitative agreement with the experimental curves is seen.

### Spontaneous particle creation in the first quench

**Fig. 3:**
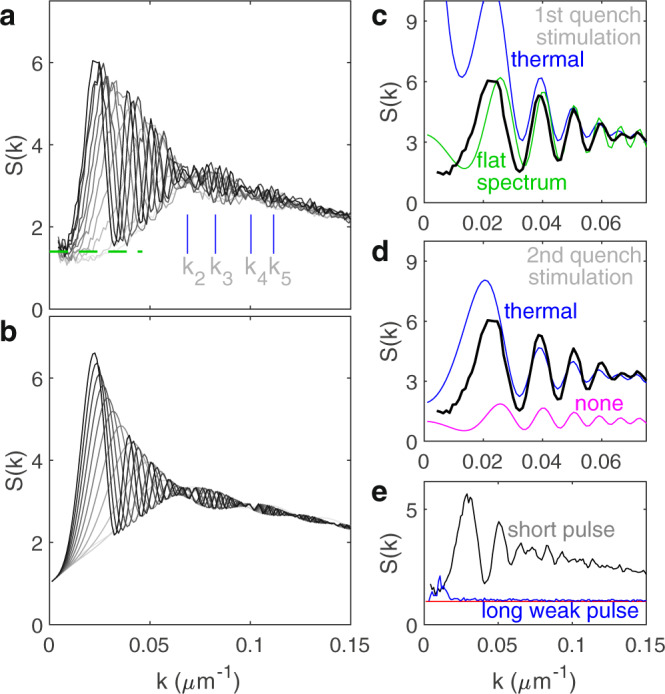
Spontaneous and stimulated cosmological particle production. **a** The envelope of *S*(*k*). The black curves of Fig. 2b are among the curves shown. Darker gray indicates later time. The low-*k* limit is indicated by the green dashed line. The *k*_*p*_ mark the nodes and antinodes. **b** The envelope of the theoretical curves. **c** The effect of stimulation in the first quench, on *S*(*k*) after both quenches. The blue curve includes additional stimulation by a thermal distribution in the first quench. The green curve includes stimulation by a flat distribution in the first quench rather than the second. *τ* = 153 ps is shown. **d** The effect of stimulation in the second quench. The blue curve includes additional stimulation by a thermal distribution in the second quench. The magenta curve includes no extra stimulation in either quench. *τ* = 153 ps is shown. **e** Effect of the interactions. The black curve is from Fig. 2b. The blue curve employs a pulse which is 500 times weaker and longer. The red curve is the theoretical prediction for the long, weak pulse.*τ* = 87 ps is shown.

The low-$k$ behavior of $S\left(k\right)$ provides a window into the early times before the quenches, since the frequency of these modes approaches zero, so the modes do not have sufficient time to evolve during the experiment. The first peak in $S(k)$ corresponds to the frequency $1/4\tau$, the lowest frequency which has time to oscillate. Well below this $k$-value, Eq. (2) reduces to $S\left(k\right)=1+2{N}_{1}$, where ${N}_{1}$ is the incoherent population before the first quench, and the unity term corresponds to the quantum shot noise, which is scale invariant (independent of $k$). Thus, the value of $S\left(k\right)$ gives a direct measure of ${N}_{1}$. Figure 3a shows the $S(k)$ curves for all $\tau$ plotted together. We observe that $S(k)$ is at most 1.4 for low $k$, as indicated by the dashed green line, giving ${N}_{1}\le$ 0.2. This value is finite and approximately scale invariant, which implies a negligible thermal component, since a thermal population diverges like $1/k$. Furthermore, it is less than unity, implying that the spontaneous contribution dominates. Thus, the analogue cosmological particle creation is spontaneous in the first quench. This is verified by the blue and green curves in Fig. 3c, which show that stimulation in the first quench by thermal noise and white noise, respectively, would produce larger values of $S\left(k\right)$ than those of the experiment, for low $k$.

### Stimulated particle creation in the second quench

The quasiparticles spontaneously created during the first quench stimulate pair creation in the second quench. However, if the particle production in the second quench were stimulated by the first-quench quasiparticles only, $S\left(k\right)$ would oscillate about unity, as indicated by the magenta curve in Fig. 3d. Rather, $S(k)$ features an upward shift relative to unity, and a downward slope for large $k$. The downward slope is due to the finite resolution of the imaging system, measured to be 10 µm (see Measuring the imaging resolution in Supplementary Methods), and is included in the theoretical curves. The upward shift results from absorption and spontaneous reemission of photons from the atomic medium. By the first two terms of Eq. (2), $S(k)$ oscillates about the value $1+2\left({N}_{1}+{N}_{{{{{{\rm{b}}}}}}}\right)$, where ${N}_{{{{{{\rm{b}}}}}}}$ is the background population present in the fluid between the two quenches. The population spontaneously created in the first quench does not contribute to this expression, since its spectrum (given by ${\beta }^{2}$ in Eq. (3)) does not extend to large $k$. The upward shift in Fig. 3a suggests ${N}_{{{{{{\rm{b}}}}}}}$ = 1.2, a value which agrees well with our estimate for spontaneous reemission (see Absorption and spontaneous reemission in Supplementary Methods). The theoretical curves in Fig. 2b include this additional stimulation. While this incoherent, flat spectrum of 1.2 quasiparticles per mode implies that the fluid is not in its ground state, like a finite-temperature Bose-Einstein condensate, it does not negate the oscillatory behavior of $S\left(k\right)$, and it even enhances the visibility of the oscillations. We can control this population by tuning the atomic density, the pulse duration, intensity, and detuning. In Fig. 3e we verify that this population vanishes for long weak pulses as expected, due to the finite coherence time of the spontaneous reemission. The unity value of $S\left(k\right)$ confirms that the fluid is shot-noise limited, when the effect of the atomic medium is absent.

Although our fluid of light is not in thermal equilibrium between the two quenches, we can put an upper limit on the effective temperature of the thermal component before the second quench. The blue curve in Fig. 3d includes thermal stimulation with an effective temperature 2$m{c}_{{{{{{\rm{s}}}}}}}^{2}$ = 30 mK, where ${c}_{{{{{{\rm{s}}}}}}}$ is the speed of sound for the Bogoliubov quasiparticles, which results in a greatly enhanced first peak. Since this enhanced peak is absent from the experimental curve, we estimate the effective temperature of the thermal component to be less than 2$m{c}_{{{{{{\rm{s}}}}}}}^{2}$, as in an atomic Bose-Einstein condensate. For the second quench, the thermal component does not diverge like $1/k$ since the zero-temperature static structure factor in the fluid of light goes to zero for low $k$ (Ref. ^31^).

### Interference pattern and the dispersion relation

**Fig. 4:**
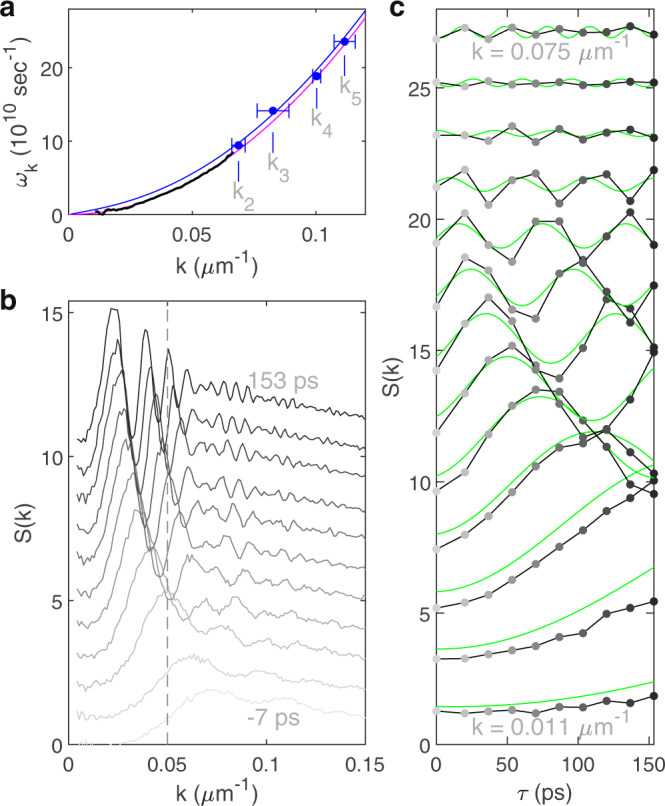
Individual modes of the analogue cosmological particles. **a** The dispersion relation. The blue points are derived from the *k*_*p*_ in Fig. 3a. The error bars indicate one standard deviation. The black curve is obtained by sinusoidal fits to the gray curves in (**c**). The magenta curve is the free-particle dispersion relation. The blue curve is the dispersion relation in the interacting fluid of light. **b** The static structure factor at various times. The curves are from Fig. 3a, and are shifted vertically. The vertical dashed line is used to find the values in (**c**). **c** Each curve shows the *τ*-dependence of a definite *k*, given by the values along a vertical line in **b**, such as the dashed line. The grayscale is the same as in (**b**). The *k*-values shown are equally spaced by 5.4 × 10^-3^ µm^-1^. The green curves are computed with Eq. (2). Each pair of black and green curves has been shifted vertically.

Figure 3a exhibits a beating pattern in the envelope of the various curves, resulting from interference between analogue cosmological particles created in the two quenches. The theoretical curves in Fig. 3b show a similar pattern. The envelope has nodes and antinodes at ${{\omega }_{12}}_{{k}_{p}}=\pi p/2{\tau }_{12}$, where $p$ is an integer (see Beating pattern in Supplementary Methods). By identifying each ${k}_{p}$ as shown in Figs. 3a, 4 points on the dispersion relation are found, as indicated by blue points in Fig. 4a. These points agree well with the dispersion relation in the medium, calculated from the interactions, and indicated by the blue curve.

### Individual modes

Figure 4b shows the curves of Fig. 3a, one above the other. By plotting the $S(k)$ values along the dashed line, we obtain the time dependence of a given mode $k$, as shown in Fig. 4c. Each mode is seen to oscillate sinusoidally after the second quench, with no visible damping. The frequencies of the oscillations, indicated by the black curve in Fig. 4a, agree well with the free-particle spectrum, which is relevant after the second quench.

### Comparison with the CMB power spectrum

**Fig. 5:**
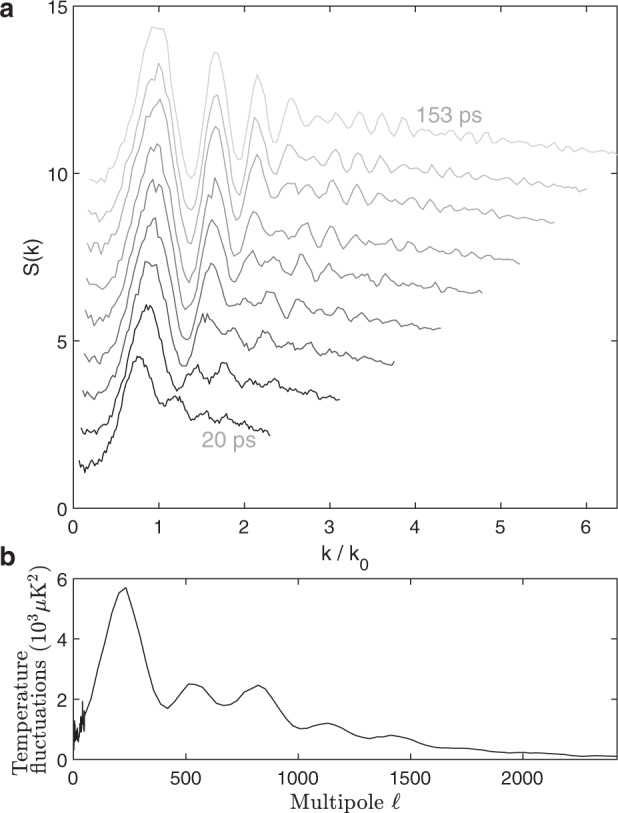
Comparing the measured power spectrum with that of the CMB. **a** The static structure factor for *τ*  > 0. The wavenumbers are normalized such that unity can be compared with the first peak of the CMB spectrum in (**b**). **b** The power spectrum of temperature fluctuations in the CMB, as a function of multipole number, from Ref. ^36^.

We compare and contrast our observed spectra with the CMB power spectrum in Fig. 5. Since 1990, several successive space missions have improved the resolution of the CMB measurements^32–35^, and Fig. 5b shows the latest results^35^. In the CMB spectrum, the oscillations occur as a result of the well-defined phase between the cosmological particles^36,37^, which is also true for our spectra. In the early universe, the density fluctuations relevant for the acoustic peaks oscillated until the effective time of observation $\tau$, when the photons decoupled from matter^38^. The mode number ${{{{{\mathcalligra{l}}}}}}$ shown in Fig. 5b is proportional to $k$, when mapped back to the density fluctuations in the early universe, and the first peak likely corresponds to ${\omega }_{k}\tau \approx \pi$ (Refs. ^39–41^). In contrast, the Bogoliubov transformation predicts a first peak in the CMB spectrum at ${\omega }_{k}\tau \approx \pi /2$ (Ref. ^5^). Figure 5a shows our spectra for $\tau > 0$. For visual comparison with the CMB spectrum, $k$ is divided by ${k}_{0}$, which satisfies ${\omega }_{{k}_{0}}\tau =\pi /2$, where ${\omega }_{k}$ is the magenta curve of Fig. 4a. There are features which are common to our spectra and that of the CMB, in addition to the oscillations: the decay of the oscillations for large $k$ or ${{{{{\mathcalligra{l}}}}}}$, and the approximately scale-invariant region for small $k$ or ${{{{{\mathcalligra{l}}}}}}$. The oscillations in the CMB spectrum decay for large ${{{{{\mathcalligra{l}}}}}}$ due to damping by photon diffusion^39^. In contrast, the oscillations in our spectra decay because the $\beta$ Bogoliubov coefficient in Eq. (4) decreases for high $k$. The scale-invariant region of the CMB spectrum arises from quantum fluctuations^42–44^, assuming that the inflation model is correct^45–48^. Similarly, the scale invariant part of our spectra reflects the quantum nature of the particle production, as a result of the vacuum of incoming particles. However, our incoming vacuum is a property of our shot-noise limited laser, as opposed to red-shifting of the modes which possibly occurred during inflation^42,49,50^. Red-shifting was observed in the laboratory^51^, and it would be interesting to combine it with analogue cosmological particle production.

## Discussion

This work establishes the paraxial fluid of light as a quantum fluid. The results demonstrate that quantum field theory applies to a system in which a spatial coordinate plays the role of time. The effective temperature is less than twice the interaction energy, which is comparable to many atomic Bose-Einstein condensates. On the other hand, the apparatus is an order of magnitude simpler, smaller, and less expensive. The direct detection of the photon fluid is also an advantage.

In conclusion, we observe both spontaneous and stimulated analogue cosmological particle creation in a quantum fluid of light. The particle production in the first quench is seen to be spontaneous, while the second includes stimulation by the first quench quasiparticles, as well as by an incoherent background. We quantitatively confirm the quantum field-theoretical prediction. The long wavelength part of the spectrum provides a window into early times before the particle creation. From an alternative perspective, we observe the spontaneous and stimulated dynamical Casimir effects in a quantum fluid.

## Methods

### Determination of $\triangle n$

The interaction between photons is quantified by the nonlinear contribution to the index of refraction $n$, given by $\triangle n=n\left(I\right)-n(0)$. We would like to express $\triangle n$ in terms of easily measurable quantities. The index of refraction is given by $n=\sqrt{1+{{{{{\rm{Re}}}}}}\left(\chi \right)}$, where $\chi$ is the atomic susceptibility. Since $n\approx 1$, one obtains $\triangle n=\triangle \left[{{{{{\rm{Re}}}}}}\left(\chi \right)\right]/2$. Furthermore, the absorption coefficient is given by ${\alpha }_{{{{{{\rm{a}}}}}}}={k}_{0}{{{{{\rm{Im}}}}}}\left(\chi \right)$. Thus,

6 $$\triangle n=\frac{{\alpha }_{{{{{{\rm{a}}}}}}}}{2{k}_{0}}\frac{\triangle \left[{{{{{\rm{Re}}}}}}\left(\chi \right)\right]}{{{{{{\rm{Im}}}}}}\left(\chi \right)}$$

Also, $\chi$ is proportional to $\left(i-2{{{{{\rm{\delta }}}}}}/\Gamma \right)\,{\left[1+{\left(2{{{{{\rm{\delta }}}}}}/\Gamma \right)}^{2}+I/{I}_{{{{{{\rm{sat}}}}}}}\right]}^{-1}$, where ${{{{{\rm{\delta }}}}}}$ is the detuning from resonance, $\Gamma$ is the linewidth, and ${I}_{{{{{{\rm{sat}}}}}}}$ is the saturation intensity. This gives

7 $$\triangle n=\frac{{\alpha }_{{{{{{\rm{a}}}}}}}}{2{k}_{0}}\frac{-\,\frac{\frac{2{{{{{\rm{\delta }}}}}}}{\Gamma }}{1+{\left(\frac{2{{{{{\rm{\delta }}}}}}}{\Gamma }\right)}^{2}+\frac{I}{{I}_{{{{{{\rm{sat}}}}}}}}}\,+\,\frac{\frac{2{{{{{\rm{\delta }}}}}}}{\Gamma }}{1+{\left(\frac{2{{{{{\rm{\delta }}}}}}}{\Gamma }\right)}^{2}}}{\,\frac{1}{1+{\left(\frac{2{{{{{\rm{\delta }}}}}}}{\Gamma }\right)}^{2}+\frac{I}{{I}_{{{{{{\rm{sat}}}}}}}}}}$$

The detuning is ${{{{{\rm{\delta }}}}}}$ = −1.5 GHz relative to the ^85^Rb cooling transition, and the self-broadened linewidth is $\Gamma /2{{{{{\rm{\pi }}}}}}$ = 16 MHz (Ref. ^52^, for the vapor cell temperature of 150 °C, corresponding to an atomic density of 1 × 10^20^ m^−^^3^. The intensity is given by $I=2P/\pi {w}^{2}$, where the waist of the beam is $w$ = 4 mm, and the laser power $P$ decays exponentially due to absorption, from 100 mW at the entrance to the vapor cell, to 20 mW at the exit. We estimate ${I}_{{{{{{\rm{sat}}}}}}}$ to be the far-detuned, $\pi$-polarized value, 25 Wm^−2^ (Ref. ^53^. The absorption coefficient is given by ${\alpha }_{{{{{{\rm{a}}}}}}}=-\left({{{{{\rm{ln}}}}}}T\right)/L$, where $T$ = 0.2 is the transmission through the vapor cell of length $L$ = 10 mm. The wavenumber is given by ${k}_{0}=2\pi /\lambda$, where $\lambda$ = 780 nm. Equation (7) yields $\triangle n$ = −8.6 × 10^−6^ and −1.7 × 10^−6^ for the entrance and exits of the vapor cell, respectively. We have neglected the effect of optical pumping into the dark ground state. Via measurements of $\triangle n$ and ${\alpha }_{{{{{{\rm{a}}}}}}}$, we find the optical pumping time to be a few microseconds, so optical pumping is negligible during the 100 ns pulse employed in this work.

### Computation of $S({k}_{x},{k}_{y},{k}_{z{\prime} }=0)$

The power spectrum (static structure factor) $S({k}_{x},{k}_{y},{k}_{z{\prime} })$ of a system of $M$ particles (photons in our case) is given by

8 $$S\left({k}_{x},{k}_{y},{k}_{z{\prime} }\right)=\frac{\left\langle {\left|\delta \rho \left({k}_{x},{k}_{y},{k}_{z{\prime} }\right)\right|}^{2}\right\rangle }{M}$$

where $\delta \rho ({k}_{x},{k}_{y},{k}_{z^{\prime}})=\int {dx}\,{dy}\,{d}{{z}^{\prime}} \,\delta \rho (x,\,y,{z^{\prime}} )\,{e}^{-i({k}_{x}x+{k}_{y}y+{k}_{z^{\prime}}{z^{\prime}})}$ and the density fluctuation $\delta \rho ({x},{y},{{z}^{\prime}})=\rho ({x},{y},{{z}^{\prime}} )-\langle \rho ({x},{y},{{z}^{\prime}})\rangle$. Setting ${k}_{z{\prime} }=0$, one obtains the 2-dimensional Fourier transform

9 $$\delta \rho \left({k}_{x},{k}_{y},{k}_{z{\prime} }=0\right)=\int {dx}\,{dy}\,\delta \widetilde{\rho }\left(x,\,y\right)\,{e}^{-i\left({k}_{x}x+{k}_{y}y\right)}$$

where $\widetilde{\rho }\left(x,\,y\right)=\int {d}{{z}^{\prime}} \rho \left(x,\,y,{z^{\prime}} \right)$ is the number density integrated in the $z{\prime}$ direction, a quantity we measure directly on the camera.

The density fluctuation $\delta \widetilde{\rho }=\widetilde{\rho }-{\left\langle \widetilde{\rho }\right\rangle }_{5}$ is computed for each image, where ${\left\langle \widetilde{\rho }\right\rangle }_{5}$ is the average of 5 adjacent images rather than the average $\left\langle \widetilde{\rho }\right\rangle$ over the entire ensemble. This technique reduces the effects of drifts in the experimental parameters during the 7 s required to obtain the ensemble. As mentioned in relation to Fig. 2c, the relative density fluctuation $\delta \widetilde{\rho }/\left\langle \widetilde{\rho }\right\rangle$ is on the order of 10^−3^, so small drifts can play a role. For example, thermal convection of the ^85^Rb gas may induce small changes in the shape of the fluid of light from image to image. The 2-dimensional Fourier transform in Eq. (9) is computed for each image within the dashed square of Fig. 1c. The power spectrum $S({k}_{x},{k}_{y},{k}_{z{\prime} }=0)$ is computed by Eq. (8). The use of ${\left\langle \widetilde{\rho }\right\rangle }_{5}$ rather than $\left\langle \widetilde{\rho }\right\rangle$ reduces the fluctuations by a factor of 4/5. Thus, $S({k}_{x},{k}_{y},{k}_{z{\prime} }=0)$ is multiplied by 5/4 to correct this effect. Furthermore, the finite quantum efficiency $Q$ = 0.485 of the camera tends to randomize the photon density and bring $S({k}_{x},{k}_{y},{k}_{z{\prime} }=0)$ closer to unity. Thus, $S({k}_{x},{k}_{y},{k}_{z{\prime} }=0)-1$ is multiplied by the factor $1/Q$.

## Supplementary information

Supplementary Information

## Data Availability

Source data are provided with this paper. The images generated in this study have been deposited in Zenodo (10.5281/zenodo.6438403). Source data are provided with this paper.

## Source data

Source Data
