# Supplementary material for: Analogue cosmological particle creation in an ultracold quantum fluid of light
Source: Nat Commun. 2022 May 25;13:2890. doi: 10.1038/s41467-022-30603-1 (PMC9133100; doi:10.1038/s41467-022-30603-1)
Supplement: Supplementary file 1 — Supplementary Information [file 41467_2022_30603_MOESM1_ESM.pdf]

Analogue cosmological particle creation in an ultracold quantum fluid of light

Jeff Steinhauer[1,2], Murad Abuzarli[1], Tangui Aladjidi[1], Tom Bienaimé[1], Clara Piekarski[1], Wei Liu[1], Elisabeth Giacobino[1], Alberto Bramati[1], and Quentin Glorieux[1]

[1]*Laboratoire Kastler Brossel, Sorbonne Université, CNRS, ENS-PSL Research University, Collège de France, Paris 75005, France*

[2]*Department of Physics, Technion—Israel Institute of Technology, Technion City, Haifa 32000, Israel*

Supplementary Methods

3-dimensional Gross-Pitaevskii equation

It is known that the wave equation for a fluid of light takes the form of the following nonlinear Schrödinger equation, within the usual paraxial and slowly-varying envelope approximations[1-3]

$$i\frac{\partial \mathcal{E}}{\partial z} = -\frac{1}{2k_0}\nabla_\perp^2\mathcal{E} + \frac{D_0}{2}\frac{\partial^2\mathcal{E}}{\partial t^2} - \frac{i}{v_\mathrm{g}}\frac{\partial \mathcal{E}}{\partial t} + U(\mathbf{r},\mathrm{t})\mathcal{E} + g(\mathbf{r},\mathrm{t})|\mathcal{E}|^2\mathcal{E} \quad \text{(S1)}$$

where $\mathcal{E}(x,y,z,t)$ is the electric field, k_0 is the wavenumber of the light, $\nabla_\perp \equiv (\partial_x, \partial_y)$, $D_0 = \partial^2 k_0/\partial\omega^2$ is the group velocity dispersion, $v_\mathrm{g} = 0.007c$ is the group velocity, $U(\mathbf{r},\mathrm{t})$ is the external potential due to an applied index of refraction, and $g(\mathbf{r},\mathrm{t})$ is the effective coupling constant due to nonlinearity. The role of time is played by the spatial coordinate z in the direction of propagation, and the fluid is 2-dimensional in the transverse coordinates x and y. Equation (S1) also contains first and second derivatives with respect to the true time t, but these are often neglected (the monochromatic approximation). However, the 3-dimensional nature of our study requires a different approach.

Firstly, we write Eq. (S1) in energy units, as in the usual Gross-Pitaevskii equation,

$$i\hbar c\frac{\partial \psi}{\partial z} = -\frac{\hbar^2}{2m}\nabla_\perp^2\psi - \frac{\hbar^2}{2m_{z\prime}v_\mathrm{g}^2}\frac{\partial^2\psi}{\partial t^2} - \frac{i\hbar c}{v_\mathrm{g}}\frac{\partial \psi}{\partial t} + U(\mathbf{r},\mathrm{t})\psi + g(\mathbf{r},\mathrm{t})|\psi|^2\psi \quad \text{(S2)}$$

where $\psi(x,y,z,t)$ is the macroscopic wavefunction, the effective photon mass is $m = \hbar k_0/c = 2.8\times10^{-36}$ kg, and $m_{z\prime} = -\hbar/cv_\mathrm{g}^2 D_0$. The mean-field interaction energy (the chemical potential) is given by $g(\mathbf{r},\tau)|\psi|^2 = -\hbar c k_0\,\Delta n/n$, and the external potential is given by $U(\mathbf{r},\tau) =$

$-\hbar c k_0 \delta n/n$, where δn is an applied change in the index of refraction. Furthermore, the healing length is given by $\xi = \hbar/mc_s$ = 60 μm, where $c_s = \sqrt{g(\mathbf{r},\tau)\rho/m} = c\sqrt{-\Delta n/n}$. The length scale associated with group velocity dispersion is given by $\xi_{z'} = \xi(m/m_{z'})^{1/2}$ = 6 mm (Ref. 1). In order for Eq's. (S1) and (S2) to be valid, the frequency interval $v_{\mathrm{g}}/2\pi\xi_{z'}$ = 50 MHz should be much less than the 1.5 GHz detuning, which is indeed the case.

Secondly, rather than neglecting the time derivatives, we make the coordinate transformation $(z,t) \rightarrow (z',\tau)$, where the effective time is $\tau = z/c$ (z plays the role of time), and

$$z' \equiv \sqrt{\frac{m_{z'}}{m}}\left(v_{\mathrm{g}} t - z\right) \tag{S3}$$

where the first factor is γ in the main text. This coordinate z' is comoving with the light at the group velocity, and is compressed due to group velocity dispersion. The relationship between z and z' is illustrated in Fig. S1. The square pulse in Fig. S1a is stationary and compressed when plotted versus z', as shown in Fig. S1b. The second factor in Eq. (S3) is from Ref. 27. The transformation is

$$\begin{pmatrix} \frac{\partial\psi}{\partial z} \\ \frac{\partial\psi}{\partial t} \end{pmatrix} = \begin{pmatrix} \frac{\partial z'}{\partial z} & \frac{\partial\tau}{\partial z} \\ \frac{\partial z'}{\partial t} & \frac{\partial\tau}{\partial t} \end{pmatrix} \begin{pmatrix} \frac{\partial\psi}{\partial z'} \\ \frac{\partial\psi}{\partial\tau} \end{pmatrix} = \begin{pmatrix} -\sqrt{\frac{m_{z'}}{m}} & \frac{1}{c} \\ v_{\mathrm{g}}\sqrt{\frac{m_{z'}}{m}} & 0 \end{pmatrix} \begin{pmatrix} \frac{\partial\psi}{\partial z'} \\ \frac{\partial\psi}{\partial\tau} \end{pmatrix} \tag{S4}$$

Applying the transformation twice gives

$$\frac{\partial^2\psi}{\partial t^2} = v_{\mathrm{g}}^2\left(\frac{m_{z'}}{m}\right)\frac{\partial^2\psi}{\partial z'^2} \tag{S5}$$

Inserting $\partial\psi/\partial z$, $\partial\psi/\partial t$, and $\partial^2\psi/\partial t^2$ from Eq's. (S4) and (S5) into Eq. (S2) gives Eq. (1), the 3-dimensional Gross-Pitaevskii equation. All three space coordinates x, y, and z' have the same mass m and healing length ξ. Thus, we have recast Eq. (S1) into the 3-dimensional Gross-Pitaevskii equation without any further approximation. The mapping carries on to the quantized field, as shown in Ref. 1. In Eq. (1), ψ is normalized such that the mean of $|\psi|^2$ is the photon density $\rho = \left(I/\hbar c k_0 v_{\mathrm{g}}\right)(m/m_{z'})^{1/2}$, where I is the laser intensity. Averaged over the dashed square in Fig. 1c, $\rho =$ 7×10^{16} m^{-3} at the exit of the vapor cell. Also, $g(\mathbf{r},\tau) = -(\hbar c k_0)^2 n_2 v_{\mathrm{g}} (m_{z'}/m)^{1/2}/n$, where n_2 is the nonlinear refractive index ($\Delta n = n_2 I$). The effective s-wave scattering length is $a_{\mathrm{s}} = mg(\mathbf{r},\tau)/4\pi\hbar^2$ = 6×10^{-11} m.

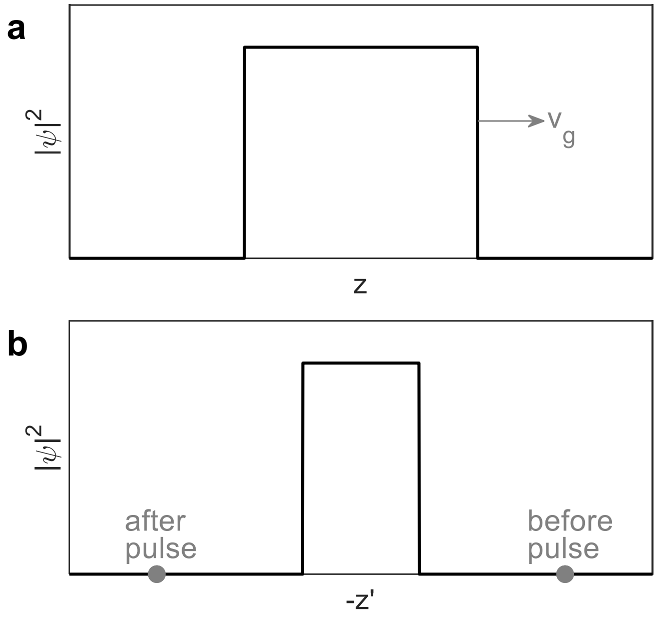

Fig. S1 The comoving and compressed coordinate. **a** A square pulse as a function of z. **b** The same pulse as a function of z'.

Absorption and spontaneous reemission

The absorption of the laser pulse implies that photons were scattered. The goal of this section is to compute the number $N_{\mathrm{m}}(t)$ of coherent photons per mode, since these photons stimulate analogue cosmological particles in the second quench.

Due to the finite coherence time $\Delta t = 1/\Gamma$ of the spontaneous reemission, the instantaneous coherent population $N_{\mathrm{m}}(t)$ is less than the integrated number of photons $N_{\mathrm{t}}(t)$ emitted into the mode before time t. The number $N_{\mathrm{t}}(t)$ will be useful since it can be determined from the absorption. The coherent population can be determined from the following rate equation

$$\dot{N}_{\mathrm{m}} = R - \frac{N_{\mathrm{m}}}{\Delta t} \tag{S6}$$

where the second term accounts for the effective loss of photons from the mode, due to decoherence. The rate R will be determined below, from $N_{\mathrm{t}}(t)$. The solution of Eq. (S6) is

$$N_{\mathrm{m}}(t) = \mathrm{R}\Delta t\left(1 - e^{-t/\Delta t}\right). \tag{S7}$$

The time average over the pulse of length t_{p} is given by

$$\bar{N}_{\mathrm{m}} = \mathrm{R}\Delta t\left\{1 - \frac{\Delta t}{t_{\mathrm{p}}}\left[1 - e^{-t_{\mathrm{p}}/\Delta t}\right]\right\}. \tag{S8}$$

Eq. (S8) gives the desired quantity, in terms of R. Since $N_t(t)$ is not sensitive to decoherence, it obeys

$$\dot{N}_t = R \tag{S9}$$

Integrating Eq. (S9) over t_p yields

$$N_t(t_p) = R t_p \tag{S10}$$

The measured absorption gives another expression for $N_t(t_p)$, in which all quantities are known,

$$N_t(t_p) = \frac{\eta}{n_m} \frac{P_L t_p}{\hbar c k_0} (1 - T) \tag{S11}$$

The factors other than the first ratio give the total number of photons removed from the pulse by absorption, where $P_L = P\ \mathrm{erf}^2(L_s/\sqrt{2}w)$ is the laser power within the dashed square in Fig. 1c, with dimension L_s = 6 mm. The total number of modes is $n_m = 4\pi k_0^2/\delta k^2 = 4\pi L_s^2/\lambda^2$, where the width of a mode is given by $\delta k = 2\pi/L_s$. The factor η accounts for the cases where the emitted photon is not of the same frequency as the absorbed photon, due to atoms which did not return to the same hyperfine F, m_F level after the absorption and reemission. Since these photons do not have the same frequency as the fluid of light, they do not become quasiparticles. By considering all possible routes from the ground state levels of ^{85}Rb, to the excited levels of the D2 line and back to the ground state, weighting each route by the matrix element squared, we find that the probability of returning to the same ground state level is η = 0.41.

The expression for R is obtained by equating Eq's. (S10) and (S11),

$$R = \frac{\eta}{n_m} \frac{P_L}{\hbar c k_0} (1 - T) \tag{S12}$$

For the t_p = 100 ns pulses used in Fig. 2, Eq's. (S8) and (S12) give $\overline{N}_m$ = 1.2. On the other hand, the blue curve in Fig. 3b employs pulses which are 500 times longer and weaker, corresponding to $\overline{N}_m$ = 0.003.

<u>The static structure factor as a measure of 2-mode fluctuations</u>

We can gain additional insight into the power spectrum by noting that it measures the relative fluctuations between two regions of the fluid of light. The vector $(k_x, k_y, k_{z'} = 0)$ defines the two areas, indicated by red and green in Fig. S2. The power spectrum is essentially given by $S(k_x, k_y, k_{z'} = 0) = \langle (N_r - N_g)^2 \rangle / \langle N_r + N_g \rangle$, where N_r and N_g are the numbers of photons in the total red and green regions. The quantity $N_r - N_g$ could be computed by multiplying the image by a square wave and summing, but sine and cosine functions are used, giving a Fourier transform.

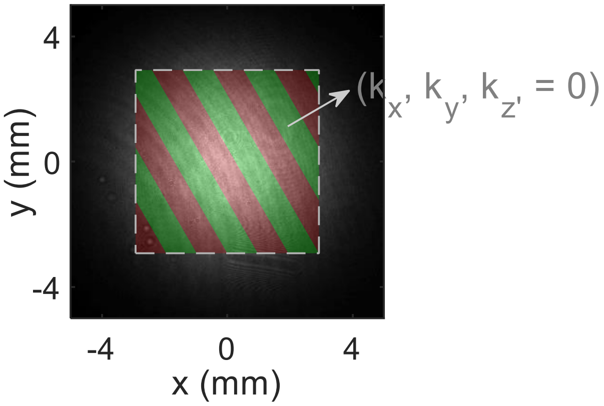

Fig. S2 The power spectrum as a measure of relative fluctuations between two areas. The total red and green regions of the image indicate the two areas defined by the vector $(k_x, k_y, k_{z\prime} = 0)$.

Measuring the imaging resolution

The resolution of the imaging system is determined from images of very small rubidium droplets on the window of the vapor cell, as shown in Fig. S3a. The sizes of the droplets are below the resolution, so the images show the point spread function of the imaging system. The Fourier transform of the images gives the response of the imaging system as a function of k. The magnitude squared of the 2-dimensional Fourier transform is computed for each image, and the average over all images is found. The azimuthal average of this quantity is shown in Fig. S3b. The dashed curve is a Gaussian fit, normalized to unity, which is taken as the response of the imaging system. The Gaussian is of the form $\exp(-R^2k^2/4)$, where R = 10 μm is the measured resolution. Throughout this work, the theoretical curves for $S(k) - 1$ are multiplied by this Gaussian.

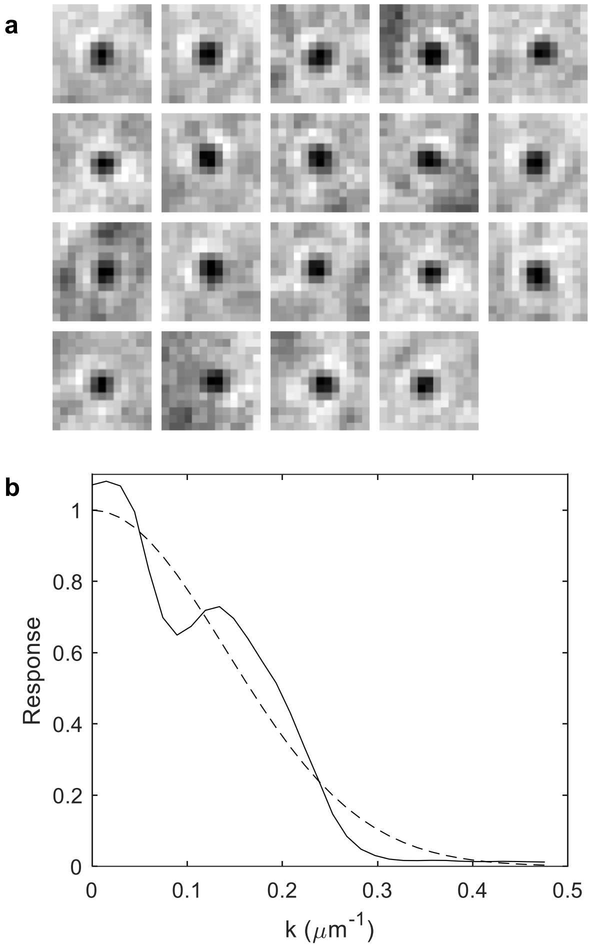

Fig. S3 The resolution of the imaging system. a Images of tiny rubidium droplets. Each image shows the point spread function of the imaging system. Each image has dimensions 85 μm × 85 μm. **b** The response of the imaging system. The solid curve is determined from the images in **a**. The dashed curve is a Gaussian fit.

$S(k)$ including absorption

The annihilation operators are given by $\hat{a}_{\mathbf{k}}$ (before the vapor cell), $\hat{b}_{\mathbf{k}}$ (in the vapor cell), and $\hat{c}_{\mathbf{k}}$ (after the vapor cell).

Before the vapor cell (the initial state)

The population is $N_1 \equiv \langle \hat{a}_{\mathbf{k}}^{\dagger} \hat{a}_{\mathbf{k}} \rangle = \langle \hat{a}_{-\mathbf{k}}^{\dagger} \hat{a}_{-\mathbf{k}} \rangle$, which may be a thermal distribution or otherwise. The population is assumed to be incoherent, so the correlations are $\langle \hat{a}_{\mathbf{k}} \hat{a}_{-\mathbf{k}} \rangle = 0$.

First quench

The first quench occurs at an effective time $\tau_0 = -L/c$. The operators before and after the quench are related by the Bogoliubov transformation $\hat{b}_{\mathbf{k}} = \alpha(\tau_0)\hat{a}_{\mathbf{k}} - \beta(\tau_0)\hat{a}^{\dagger}_{-\mathbf{k}}$, where the Bogoliubov coefficients are given by $\alpha(\tau_0) = \left(\sqrt{S_0} + 1/\sqrt{S_0}\right)/2$, and $\beta(\tau_0) = \left(\sqrt{S_0} - 1/\sqrt{S_0}\right)/2$, where $S_0 = (\xi k)^2/\sqrt{4(\xi k)^2 + (\xi k)^4}$, and ξ is the healing length just after the first quench[4]. The population after the first quench is given by $\langle \hat{b}^{\dagger}_{\mathbf{k}}\hat{b}_{\mathbf{k}}\rangle = \alpha^2(\tau_0)N_1 + \beta^2(\tau_0)(1 + N_1) + N_{\mathrm{b}}$, where the first two terms are from the Bogoliubov transformation, and N_{b} is the background distribution of quasiparticles inside the vapor cell, thermal or otherwise. The correlations after the first quench are given by $\langle \hat{b}_{\mathbf{k}}\hat{b}_{-\mathbf{k}}\rangle = -\alpha(\tau_0)\beta(\tau_0)(1 + 2N_1)$. These populations and correlations are the initial state for the second quench.

Second quench

The second quench occurs at $\tau = 0$, and the operators before and after are related by the Bogoliubov transformation $\hat{c}_{\mathbf{k}} = \alpha(0)\hat{b}_{\mathbf{k}} + \beta(0)\hat{b}^{\dagger}_{-\mathbf{k}}$, where the Bogoliubov coefficients include the effects of propagation and absorption in the vapor cell. We find that the Bogoliubov coefficients obey the wave equation[5]

$$i\frac{\partial}{\partial\tau}\begin{bmatrix}\alpha(\tau)\\ \beta(\tau)\end{bmatrix} = \mathcal{K}(\tau)\begin{bmatrix}\alpha(\tau)\\ \beta(\tau)\end{bmatrix} \tag{S13}$$

where $\mathcal{K}(\tau) \equiv \begin{pmatrix} \frac{ic\alpha_{\mathrm{a}}}{2} + \frac{\hbar k^2}{2m} + ck_0\Delta n & ck_0\Delta n \\ -ck_0\Delta n & \frac{ic\alpha_{\mathrm{a}}}{2} - \frac{\hbar k^2}{2m} - ck_0\Delta n \end{pmatrix}$

We find

$$\begin{bmatrix}\alpha(\tau)\\ \beta(\tau)\end{bmatrix} = \prod_{q=M}^{0} e^{-i\mathcal{K}(\tau_q)\delta\tau}\begin{bmatrix}\alpha(\tau_0)\\ \beta(\tau_0)\end{bmatrix} e^{c\alpha\tau/2} \tag{S14}$$

where the first two factors are the solution of Eq. (S13) from Ref. 5, the last factor is added to ensure that $|\alpha(\tau)|^2 - |\beta(\tau)|^2 = 1$, $\delta\tau = (\tau - \tau_0)/Q$, and $\tau_q = q\delta\tau$. The integer Q should be large enough to ensure convergence. Equation (S14) is evaluated at $\tau = 0$ to obtain the Bogoliubov coefficients for the second quench, $\alpha(0)$ and $\beta(0)$.

The populations after the second quench are given by $\langle \hat{c}^{\dagger}_{\mathbf{k}}\hat{c}_{\mathbf{k}}\rangle = |\alpha(0)|^2\langle \hat{b}^{\dagger}_{\mathbf{k}}\hat{b}_{\mathbf{k}}\rangle + |\beta(0)|^2\left(1 + \langle \hat{b}^{\dagger}_{\mathbf{k}}\hat{b}_{\mathbf{k}}\rangle\right) + 2\langle \hat{b}_{\mathbf{k}}\hat{b}_{-\mathbf{k}}\rangle\,\mathrm{Re}[\alpha(0)\beta^*(0)]$, which becomes

$$\langle \hat{c}^{\dagger}_{\mathbf{k}}\hat{c}_{\mathbf{k}}\rangle = |\beta(0)|^2 + [|\alpha(0)|^2 + |\beta(0)|^2][\alpha^2(\tau_0)N_1 + \beta^2(\tau_0)(1 + N_1) + N_{\mathrm{b}}] \\ -2\alpha(\tau_0)\beta(\tau_0)(1 + 2N_1)\,\mathrm{Re}[\alpha(0)\beta^*(0)] \tag{S15}$$

The correlations just after the second quench are given by $\langle \hat{c}_{\mathbf{k}} \hat{c}_{-\mathbf{k}} \rangle = [\alpha^2(0) + \beta^2(0)] \langle \hat{b}_{\mathbf{k}} \hat{b}_{-\mathbf{k}} \rangle + \alpha(0)\beta(0)\big(1 + 2\langle \hat{b}_{\mathbf{k}}^{\dagger} \hat{b}_{\mathbf{k}} \rangle\big)$, which becomes

$$\langle \hat{c}_{\mathbf{k}} \hat{c}_{-\mathbf{k}} \rangle = -[\alpha^2(0) + \beta^2(0)]\alpha(\tau_0)\beta(\tau_0)(1 + 2N_1) + \alpha(0)\beta(0)\{1 + 2[\alpha^2(\tau_0)N_1 + \beta^2(\tau_0)(1 + N_1) + N_{\mathrm{b}}]\} \quad \text{(S16)}$$

Power spectrum

In the Bogoliubov approximation, the Fourier transform of the density operator is given by $\rho_{\mathbf{k}} = \sqrt{N}[\alpha(\tau) + \beta(\tau)]\big(\hat{c}_{\mathbf{k}}^{\dagger} + \hat{c}_{-\mathbf{k}}\big)$. The power spectrum a time τ after the second quench is given by $S(k) = \langle \rho_{\mathbf{k}} \rho_{-\mathbf{k}} \rangle / N = [\alpha(\tau) + \beta(\tau)]^2 \big[1 + 2\langle \hat{c}_{\mathbf{k}}^{\dagger} \hat{c}_{\mathbf{k}} \rangle + 2\mathrm{Re}\big(\langle \hat{c}_{\mathbf{k}} \hat{c}_{-\mathbf{k}} \rangle e^{-i2\omega_k \tau}\big)\big]$, where the $\exp(-i\omega_k \tau)$ dependence of $\hat{c}_{\mathbf{k}}$ is included. Since there are no interactions after the second quench, $\alpha(\tau)$ = 1 and $\beta(\tau)$ = 0, and the power spectrum becomes

$$S(k) = 1 + 2\langle \hat{c}_{\mathbf{k}}^{\dagger} \hat{c}_{\mathbf{k}} \rangle + 2\mathrm{Re}\big(\langle \hat{c}_{\mathbf{k}} \hat{c}_{-\mathbf{k}} \rangle e^{-i2\omega_k \tau}\big)$$

where $\langle \hat{c}_{\mathbf{k}}^{\dagger} \hat{c}_{\mathbf{k}} \rangle$ and $\langle \hat{c}_{\mathbf{k}} \hat{c}_{-\mathbf{k}} \rangle$ are given by Eq's. (S15) and (S16), respectively.

Beating pattern

We can obtain a simple expression for the beating seen in Figs. 3a and 3b by neglecting absorption and approximating $\alpha \approx 1$ and $\beta \ll 1$, which is valid for all but the lowest values of k. This yields

$$S(k) = 1 + 2(N_1 + N_{\mathrm{b}}) + 4\beta N_{\mathrm{b}} cos(2\omega_k \tau) + 4\beta(1 + 2N_1)\sin\big(\omega_{12_k}\tau_{12}\big)\sin\big(2\omega_k \tau + \omega_{12_k}\tau_{12}\big) \quad \text{(S17)}$$

where $\omega_{12_k} = \sqrt{g\rho k^2/m + (\hbar k^2/2m)^2}$ and $\omega_k = \hbar k^2/2m$ are the Bogoliubov frequencies between the quenches and after the second quench, respectively, and τ_{12} is the time difference between the quenches. The last term in Eq. (S17) results from the interference. The $\sin\big(\omega_{12_k}\tau_{12}\big)$ factor is the envelope, which has nodes and antinodes at $\omega_{12_{k_p}} = \pi p / 2\tau_{12}$, where p is an integer.

1. Larré P.-É. & Carusotto, I. Propagation of a quantum fluid of light in a cavityless nonlinear optical medium: General theory and response to quantum quenches. *Phys. Rev. A* **92**, 043802 (2015).
2. Carusotto, I. Superfluid light in bulk nonlinear media. *Proc. R. Soc. A* **470**, 20140320 (2014).
3. Boyd, R. W. *Nonlinear optics* (Elsevier, 2003).
4. Bogolubov, N. On the theory of superfluidity. *J. Phys.* **11**, 23-32 (1947).
5. Larré, P.-É., Biasi, S., Ramiro-Manzano, F., Pavesi, L. & Carusotto, I. Pump-and-probe optical transmission phase shift as a quantitative probe of the Bogoliubov dispersion relation in a nonlinear channel waveguide. *Eur. Phys. J. D* **71**, 146 (2017).
